# Trends in vaping and smoking following the rise of disposable e-cigarettes: a repeat cross-sectional study in England between 2016 and 2023

**DOI:** 10.1016/j.lanepe.2024.100924

**Published:** 2024-05-23

**Authors:** Harry Tattan-Birch, Jamie Brown, Lion Shahab, Emma Beard, Sarah E. Jackson

**Affiliations:** a Department of Behavioural Science and Health, University College London, London, UK; b SPECTRUM Consortium, UK

**Keywords:** Vaping, Electronic nicotine delivery systems, ENDS, Single-use, Tobacco

## Abstract

**Background:**

There has been a rapid rise in disposable (single-use) e-cigarette vaping among young adults in England since June 2021 (leading to a planned ban on these products). We examined how this has affected population trends in current (i) vaping, (ii) tobacco smoking, and (iii) inhaled nicotine use.

**Methods:**

We used data from a nationally-representative monthly repeat cross-sectional survey of adults (≥18) in England (n = 132,252; July-2016–May-2023). Using interrupted time-series analyses (segmented logistic regression), we estimated yearly trends in current tobacco smoking, vaping, and inhaled nicotine use (smoking and/or vaping) before (‘pre-disposables’) and after June-2021 (‘post-disposables’), stratified by age group (18 to 24, 25 to 44, 45 and over). We also examined trends in daily use and in vaping among never-smokers.

**Findings:**

Pre-disposables, vaping and smoking prevalence had been stable or declining across all age groups. However, post-disposables, the odds of current vaping increased by 99% per year among 18 to 24-year-olds (odds ratio [OR] = 1.99; 95% confidence interval [CI] = 1.71 to 2.31), compared with 39% (OR = 1.39; 95% CI = 1.26 to 1.52) in 25 to 44-year-olds and 23% (OR = 1.23; 95% CI = 1.12 to 1.35) in those aged 45 or older. Smoking rates continued to decline — albeit modestly — in 18 to 24-year-olds (OR = 0.88, 95% CI = 0.77 to 1.00) and 25 to 44-year-olds (OR = 0.93, 95% CI = 0.86 to 1.00), but increased among those aged 45 or older (OR = 1.12, 95% CI = 1.05 to 1.20). As a result, post-disposables, the overall prevalence of inhaled nicotine use increased across all age groups. Trends were similar for daily use, but post-disposables increases in vaping were greatest among people who had never regularly smoked (e.g., 18 to 24-year-olds: OR = 2.50, 95% CI = 1.82 to 3.43).

**Interpretation:**

Since disposable vapes started becoming popular in England, historic declines in nicotine use have reversed. Now, nicotine use appears to be rising, driven primarily by sharp increases in vaping among young people. Smoking declines have been most pronounced in age groups with the largest increases in vaping.

**Funding:**

10.13039/501100000289Cancer Research UK.

**Research in context**

**Evidence before this study**

We searched PubMed up to December 2023 using the following search terms (“disposable” or “single-use”) AND (“e-cigarette∗” OR “vap∗”) AND (“England” OR “Great Britain” OR “United Kingdom”) AND (“trend∗” OR “ban∗”). Three studies were identified by these searches, of which one reported data on disposable e-cigarette vaping among adults since modern disposable vapes became available. This study found that the use of disposable e-cigarette grew rapidly among young adults from May 2021 to April 2022.

**Added value of this study**

No studies have examined the potential impact of rising disposable vaping on trends in the prevalence of vaping, smoking and inhaled nicotine use among adults in Great Britain. Our results show that in England, up to June 2021, smoking and vaping had been stable or declining across all age groups. However, since disposable vaping started to become popular, vaping has been increasing across all age groups — especially younger adults (18 to 24-year-olds). Declines in smoking have stagnated or reversed in older adults (≥45) but continued among the age group with the largest increases in vaping (18 to 24-year-olds).

**Implications of all the available evidence**

Urgent action is needed to curb the rise in disposable vaping among people who would otherwise avoid nicotine entirely. This could include an excise tax, packaging restriction, and putting vapes behind shop counters. A plan to ban disposable vapes was announced by UK government in January 2024. However, policies must avoid signalling that cigarette smoking, the most harmful form of nicotine use, is a better alternative.

## Introduction

E-cigarettes use (‘vaping’) exposes people to fewer known harmful toxicants and carcinogens than cigarette use (‘smoking’)^1^ and is effective for smoking cessation.^2^^,^^3^ The UK has therefore attempted to regulate e-cigarettes proportionately, with the aim of encouraging adults to switch from smoking to vaping (e.g., through clinical guidelines)^4^ while minimising use among those who do not smoke (e.g., through pack health warnings and restrictions on advertising, nicotine concentration and age-of-sale). Up to 2021, vaping remained rare (<0.8%) in youth and adults who had never smoked.^5^ In the last three years, a new form of disposable (single-use) e-cigarette has entered the market, sold under brand names including ‘Puff Bar’, ‘Elf Bar’, and ‘Lost Mary’. These new disposable products deliver nicotine effectively using high-concentration (20 mg/ml in EU/UK) nicotine salts e-liquid, while being cheap, concealable and convenient to use.^6^ From 2021 to 2022, use of disposable vapes rose sharply in England as these new products rapidly became popular among young people.^7^^,^^8^ Similar trends were observed globally.^9^, ^10^, ^11^, ^12^ As a result of this and environmental concerns, many countries have banned the sale of disposable vapes or are reportedly considering doing so, including the UK who have announced a plan to ban these products.^13^, ^14^, ^15^, ^16^, ^17^ Understanding how the growing popularity of new disposable e-cigarettes is affecting trends in vaping and smoking is important for informing policy decisions around the regulation of these products, including potential bans, and evaluating their likely public health impact.

There are three key and interrelated questions that need to be answered. First, are new disposables displacing other types of e-cigarettes among young people who vape or are they attracting those who would otherwise not have used nicotine to take up vaping? Promotion of disposable e-cigarette brands on social media platforms such as TikTok (which is predominantly used by people aged 16 to 24^18^) is commonplace, with viral videos featuring the products being viewed by millions of users and portraying use by underage youth.^19^, ^20^, ^21^ Some of these videos are sponsored by disposable e-cigarette brands or retailers while others are shared organically (i.e., not paid for by advertisers).^20^ In both cases, it is likely that exposure to this content will influence young peoples' attitudes and behaviours.^22^ Consistent with this, qualitative data from previous studies suggest youths perceive disposable e-cigarettes as ‘cool’, ‘fashionable’ and enticing and view them as a modern lifestyle ‘accessory’, in contrast to tank vaping devices, which are perceived as being used by older adults.^23^ Population data from the Smoking Toolkit Study in Great Britain up to April 2022 suggested disposable e-cigarettes likely mostly attracted people who would otherwise use other types of e-cigarettes or cigarettes rather than non-users of any nicotine product.^7^ As the prevalence of disposable vaping rose rapidly, we detected no evidence of a rise in the percentage of young adults using any form of inhaled nicotine (i.e., vaping or smoking): while the prevalence of vaping rose significantly, there was also a potential decline in smoking.^7^ However, the Action on Smoking and Health (ASH)-Youth and ASH-Adult surveys in 2021 and 2022 showed increases among youth and young adults in England in the overall prevalence of vaping (11 to 18 y: 4.0% to 8.6%; 18 to 24 y: 5.4% to 11.0%) and potentially smoking (11 to 18 y: 4.1% to 6.0%; 18 to 24 y: 12.6% to 14.6%),^1^ suggesting a possible link between rising use of disposable vapes and increased use of inhaled nicotine among young people that urgently requires further assessment using the most up-to-date data.

The second question we need to address is whether new disposables are contributing to a change in smoking prevalence. The effect of vaping on cigarette smoking is highly contested. While randomised controlled trial evidence shows that e-cigarettes help adult smokers quit when provided alongside behavioural support,^24^ concerns have been raised about their effect on uptake of smoking among young people. Longitudinal studies consistently show that adolescents who vape are more likely to later start smoking.^25^^,^^26^ It is not widely accepted whether this association is explained by common liabilities—factors that predispose people to take up both smoking and vaping—or reflects a true causal effect of vaping leading to greater smoking in people who would otherwise avoid nicotine entirely.^27^ Some argue, based on data from time trend analyses and natural experiments, that e-cigarettes act as substitutes for cigarettes among youth, causing many who would otherwise smoke cigarettes to vape e-cigarettes instead (although a trend analysis in US adolescents and young adults by Pierce 2024^28^ does not support this).^29^, ^30^, ^31^, ^32^, ^33^ These potential effects are not mutually exclusive: it is likely that vaping causes some people to start smoking, while it stops others from doing so. The important question for public health is what is the net effect of these pathways. Examining the extent to which trends in smoking among young people have changed, and in which direction, since the rapid rise in disposable vaping can provide insight that supports development of a proportionate regulatory response that protects population health.

If new disposables are causing a rise in vaping prevalence, the final question is: how much of this is driven by experimentation and casual use versus daily use?^34^ Daily use is indicative of greater nicotine dependence on the products. This is relevant to concerns about any potential adverse impacts on health. Although it is substantially less harmful than smoking cigarettes, vaping carries risks compared with not using any nicotine product.^1^ Studies that have compared biomarkers of toxicant exposure between vapers and people who neither smoke nor vape have shown that although their exposure to many toxicants was similar, exposure to some harmful substances (e.g., acrylonitrile, a possible carcinogen^35^) was higher among vapers.^1^ Any such effects on health are likely to be lower for occasional compared with daily users. Likewise, any gateway effect on smoking uptake is less likely with occasional use, as nicotine dependence tends to be lower among youth who vape less frequently.^36^

The aim of this study is therefore to examine the extent to which the rapid rise in disposable vaping among young adults in England starting in June 2021 has affected trends in the prevalence of current and daily (i) vaping, (ii) smoking, and (iii) inhaled nicotine use. We will also consider associations with non-disposable vaping (to explore potential displacement of other device types) and vaping among never-smokers (to explore potential recruitment of tobacco-naïve users).

As the rise in disposable vaping has been much larger among younger than middle-aged and older adults, these age groups can be used to provide a control: if changes in trends in vaping and smoking outcomes are similar in older and younger age groups, despite prevalence of disposable vaping remaining relatively stable in older adults (as was the case through to mid-2022^7^), this would suggest that changes are caused by factors other than disposable vaping. If they are instead greater in younger than older adults, this adds support to the hypothesis that rises in disposable vaping cause changes in smoking. Thus, we will provide an update on age-specific trends in the prevalence of disposable vaping among adults up to May 2023, and use segmented regression (interrupted time-series) to estimate associations between the rise in disposable vaping and changes in smoking and vaping trends by age group.

## Methods

### Design

Data were drawn from the Smoking Toolkit Study, a monthly repeat cross-sectional survey that recruits a new sample of ∼1700 adults each month, which is nationally representative of the adult (aged ≥18 years) population in England. Survey weights are constructed using raking, adjusting data such that the weighted sample matched the population of England in terms of key variables including government office region. These key variables are determined each month using data from the UK Census, the Office for National Statistics mid-year estimates, and the National Readership Survey.

Interviews were initially conducted face-to-face, but the Covid-19 pandemic and associated social distancing measures led interviews after March 2020 to be performed via telephone. Both methods used a combination of quota and random location sampling (stratified by government office region) and the same weighting approach. A wave of parallel face-to-face and telephone interviews showed comparable results,^37^ but we nonetheless adjust for the change in modality to account for any potential differences. Further details of survey methods are available elsewhere.^38^^,^^39^

### Participants

We included participants who completed interviews between July 2016 (the first wave to include data on e-cigarette device usage) and May 2023 (the most recent available data at the time of analysis) inclusive. All participants gave oral informed consent and the Ethics Committee at University College approved the study (0498/001).

### Measures

#### Disposable e-cigarette vaping

Participants who reported current e-cigarette use (as defined below) were asked which type of e-cigarette device they mainly use. They could only choose one device type (the one they ‘mainly’ use). **Disposable vaping** was determined by the response, ‘a disposable e-cigarette or vaping device (non-rechargeable)’.

#### Outcomes

Smoking status was assessed by asking participants which of the following applies to them.

a. I smoke cigarettes (including hand-rolled) every day
b. I smoke cigarettes (including hand-rolled), but not every day
c. I do not smoke cigarettes at all, but I do smoke tobacco of some kind (e.g., pipe, cigar or shisha)
d. I have stopped smoking completely in the last year
e. I stopped smoking completely more than a year ago
f. I have never been a smoker (i.e., smoked for a year or more)

They were informed that the question does not refer to e-cigarettes or heated tobacco products. **Current smoking** was defined as responding *a-c* (i.e., including people who smoke cigarette and other types of tobacco). Those who responded *a* were considered to be **daily cigarette smoking** (i.e., only including people who smoke cigarettes specifically). Those who responded *f* were considered **never smokers**.

Vaping status was determined by a set of questions asking about use of a range of nicotine products. People who currently smoke were asked ‘Do you regularly use any of the following in situations when you are not allowed to smoke?’; people who currently smoke or quit in the last year were asked ‘Can I check, are you using any of the following either to help you stop smoking, to help you cut down or for any other reason at all?’; and people who do not smoke were asked ‘Can I check, are you using any of the following?’. **Current vaping** was defined as reporting using e-cigarettes (including Juul, which is included as a separate response option) in response to any of the above questions. The prevalence of nicotine pouch and heated tobacco use remains extremely rare (<0.5%) in England, so these products were not considered in this analysis.

Frequency of vaping was assessed by asking participants who vape: ‘How many times per day on average do you use your nicotine replacement product or products? If you do not use it every day, do you use it at least once a week or less often than once a week?’ **Daily vaping** was defined as reporting vaping at least once a day. Since April 2022, this variable was only measured quarterly rather than monthly so the sample size for analyses of this variable was reduced.

**Current inhaled nicotine use** was defined as reporting current smoking and/or current vaping (excluding heated tobacco products and NRT inhalators, given the low prevalence of use of these products in England), as defined above. **Daily inhaled nicotine use** was derived based on reported daily cigarette smoking and/or daily vaping, as defined above (as with daily vaping, this was only measured quarterly since April 2022).

**Non-disposable vaping** was defined as current vaping and reporting mainly using a refillable (tank/mod) or pod e-cigarette or vaping device.

#### Sociodemographic characteristics

Participants were asked to provide their exact **age** in years. Those who refused to give their exact age were asked to select their age group from a list. For all participants, age was categorised into three groups: 18 to 24, 25 to 44, and 45 or older years.

**Gender** (woman: no/yes), **occupational social grade** (ABC1 = managerial, administrative or professional occupations/C2DE = routine and manual occupation and unemployed), and **alcohol consumption** (AUDIT-C, square-rooted to approximately normalise the distribution) were included as covariates to ensure associations were not the result of temporal changes in demographics or drinking in the population.^40^^,^^41^

### Interruption

The event of interest was the rise in prevalence of disposable e-cigarette use seen among young people since June 2021, driven by the introduction and rapid adoption of new disposable vaping products.^7^ Previous data from the Smoking Toolkit Study indicate disposable e-cigarette use among 18-year-olds in Great Britain was relatively rare at <1% (<5% of those who vaped) up to May 2021, but rose gradually to 11% (55%) by April 2022.^7^ Thus, the time-series was divided into two periods: the pre-disposables period up to May 2021 and the post-disposables period from June 2021 onwards. Sensitivity analyses shifted the timing of the interruption by one and two months (i.e., July and August 2021) to check whether this affected the pattern of results.

### Analysis

Weighted logistic regression was used to provide up-to-date estimates of trends in disposable e-cigarette use among adults in England (using survey weights described earlier). Using data collected from monthly cross-sectional samples, we estimated age-specific monthly time trends in the prevalence of: (i) disposable vaping and (ii) daily disposable vaping. Models included time, age and their interaction as predictors — thus allowing for time trends to differ across ages. Time was modelled continuously using restricted cubic splines with four knots. This allowed the relationship of prevalence with time to be flexible and non-linear, while avoiding arbitrary categorisation. Age was split into three categories (18– to 24, 25 to 44, and ≥45 year-olds) to illustrate how trends differ across ages.

Segmented regression (interrupted time-series) was then used to assess the association of the rise in disposable vaping starting in June 2021 with changes in the trends of each of the following outcomes: (i) current vaping, (ii) current smoking, and (iii) current inhaled nicotine use. To do so, we used weighted logistic regression models. We modelled the trend in each outcome before the interruption (underlying secular trend; coded 1 … *n*, where *n* was the total number of waves) and the change in the trend (slope) post-relative to pre-disposables (coded 0 before the rise in disposable e-cigarette use and 1 … *m* from June 2021 onwards, where *m* was the number of waves after June 2021). Models were adjusted for seasonality (with month of the year, 0 to 12, modelled non-linearly using cyclic cubic splines)^42^ and covariates. Models also included a 0/1 indicator variable to account for the change in interview modality from face-to-face to telephone after March 2020. The analysis assumed a linear trend in our outcomes (on log-odds scale) within each time period, which was verified via visual inspection of modelled trend lines fitted alongside raw quarterly estimate (Fig. 2). Models were run separately for 18 to 24-year-olds, 25 to 44-year-olds, and those aged 45 years or older. We used predicted estimates from these models to plot pre- and post-interruption trends in the weighted prevalence of each outcome.

**Fig. 1:**
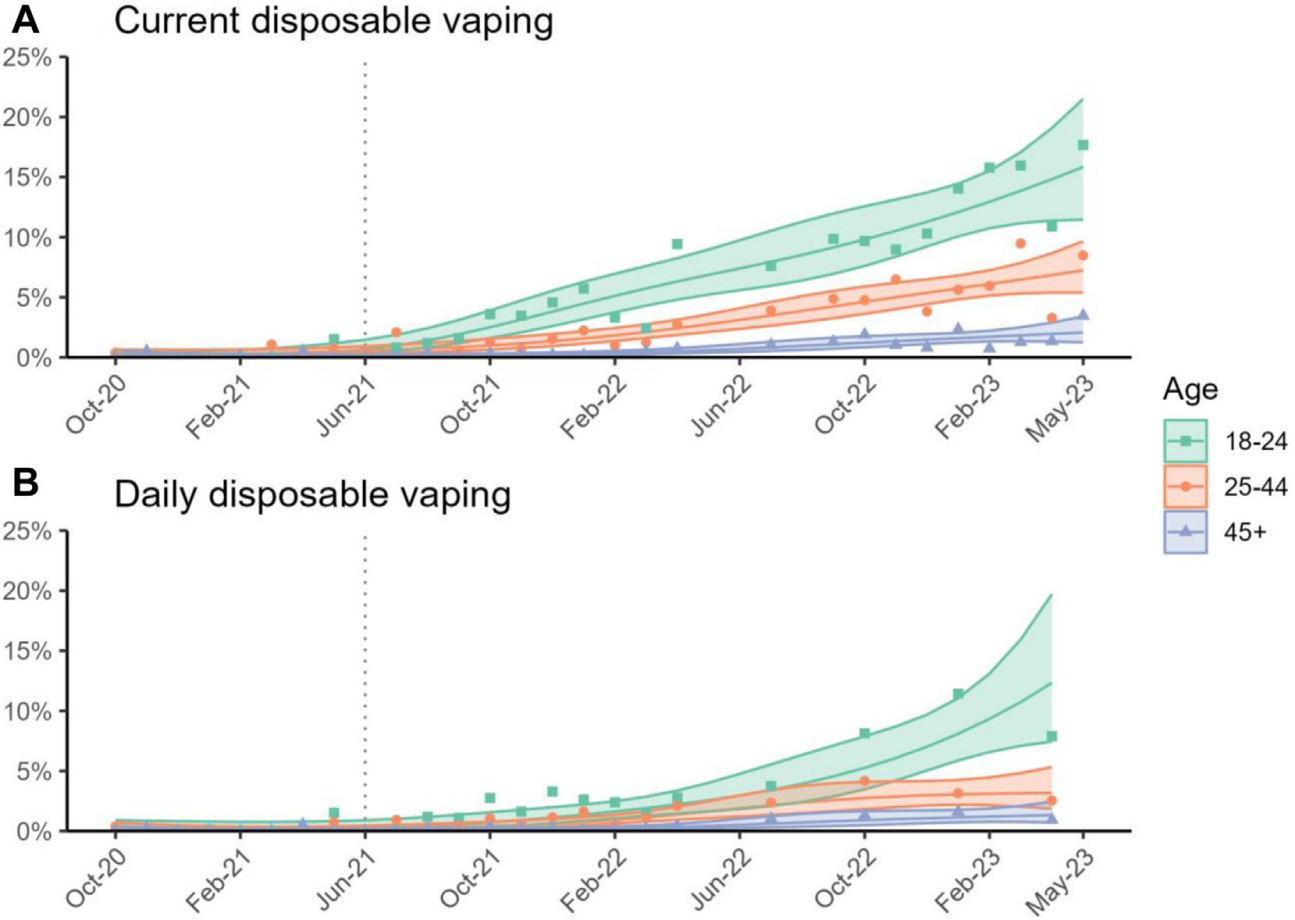
**Prevalence of (A) current and (B) daily disposable vaping among adults in England by age, October 2020 to May 2023.** Lines represent weighted point estimates from logistic regression including an interaction between age and month (modelled non-linearly using restricted cubic splines with four knots), while shaded areas represent 95% confidence intervals. Points represent weighted monthly prevalence estimates. The vertical dotted line shows the assumed interruption separating pre- and post-disposable periods. Disposable vaping was not measured in May, June, or August 2022 and daily disposable vaping was only measured quarterly from April 2022 onwards.

We ran four sensitivity analyses on the segmented regression models. The first shifted the timing of the interruption later by one and two months (i.e., July and August 2021). The purpose of this was to check whether — given the growth in disposable vaping occurred over the space of two years, starting during spring/summer 2021 — the exact month selected for the interruption (in spring/summer 2021) affected the pattern of results. The second restricted the outcomes to daily use. The third repeated the model for current vaping, restricted to use of non-disposable vapes, to explore whether the rise in disposable vaping was associated with a fall in non-disposable vaping (which would suggest a displacement effect). The fourth repeated the model for current vaping restricting the outcome to current vaping among never-smokers to explore a potential association with uptake of vaping among people who have never regularly smoked.

Finally, we modelled the changes in outcomes associated with the prevalence of disposable vaping as a continuous variable rather than an abrupt interruption. To do so, we used aggregate weighted prevalence estimates for each combination of month and age group. Then, linear regression was used to estimate the association between disposable vaping prevalence and (i) vaping prevalence, (ii) smoking prevalence, and (iii) inhaled nicotine use prevalence in the subsequent month (i.e., one-period lag), after adjustment for underlying trends, seasonality (using cyclic splines), age group, and the change in interview modality. We used Durbin Watson tests to check for autocorrelation.

Following a reviewer request, we reran primary segmented regression models stratified by (i) gender and (ii) social grade. Multiple imputation would be considered if missingness was above 5% but, because missingness was low (2.1%), analyses used data from complete cases. All analyses were performed in R version 4.1.0, and analysis code is available online (https://osf.io/z3hgc/). Authors HTB and JB directly accessed and verified the underlying data.

### Pre-registration and changes from protocol

The study protocol was registered prior to data analysis (https://osf.io/z3hgc/). There were three changes from protocol. First, as there were convergence issues when using the planned log-binomial model with random intercepts for survey month, we instead used logistic regression throughout. Second, housing tenure was not measured throughout the entire time-series, so it was not included as a covariate. Third, we originally planned to examine trends across all of Great Britian. However, the period from when Scotland/Wales started being surveyed (October 2020) to when disposables started becoming popular (June 2021) was too short to precisely estimate the pre-disposables trend or the change in trend across Great Britain. Thus, we restricted analyses to England and expanded the pre-disposables period back to July 2016, the first wave in which e-cigarette device type was measured. Analyses for Great Britain are provided online (https://osf.io/z3hgc/).

### Role of the funding source

The Smoking Toolkit Study funders were not involved in the study design or conduct; collection, management, analysis, or interpretation of data; preparation, review or approval of the manuscript; or the decision to submit the manuscript for publication. All authors were responsible for the decision to submit the manuscript.

## Results

Of the 135,114 people interviewed, 521 (0.4%) had missing data on their smoking or vaping status. An additional 2371 (1.8%) had missing data on their covariates — largely from missing alcohol consumption — leaving an analytic sample of 132,252. Sociodemographic characteristics (age, gender, social grade, and alcohol consumption) of the sample are shown in Table 1.

**Table 1:** Weighted sample characteristics.

	Pre-disposables, N = 95,409	Post-disposables, N = 36,843
**Current inhaled nicotine use**		
No	77,634 (81.4%)	29,536 (80.2%)
Yes	17,775 (18.6%)	7307 (19.8%)
**Current smoking**		
No	79,905 (83.7%)	31,192 (84.7%)
Yes	15,504 (16.3%)	5651 (15.3%)
**Current vaping**		
No	90,218 (94.6%)	33,702 (91.5%)
Yes	5191 (5.4%)	3141 (8.5%)
**Age**		
18 to 24	11,911 (12.5%)	4135 (11.2%)
25 to 44	27,094 (28.4%)	10,941 (29.7%)
≥45	56,404 (59.1%)	21,767 (59.1%)
**Female**		
No^a^	46,971 (49.2%)	18,498 (50.2%)
Yes	48,438 (50.8%)	18,345 (49.8%)
**Social grade**		
Disadvantaged (C2DE)	37,019 (38.8%)	12,075 (32.8%)
Advantaged (ABC1)	58,390 (61.2%)	24,768 (67.2%)
**Alcohol consumption (AUDIT-C)**^a^	M = 3.10 (SD = 3.04)	M = 3.50 (SD = 3.13)

Categorical variables shown as n (%); continuous variables shown as mean [M] (standard deviation [SD]). Pre-disposables period was from July 2016 to May 2021. Post-disposables period was from June 2021 to May 2023.

a For gender, 0.3% of participants identified “in another way”. AUDIT-C ranges from 0 to 12, with higher scores indicating greater alcohol consumption.

Fig. 1A shows that the rise in disposable vaping prevalence started around June 2021 and was most pronounced among the youngest age groups. Prevalence of disposable vaping in 18 to 24-year-olds increased continuously from 0.4% (95% CI = 0.2% to 1.2%) in May 2021 to 15.8% (95% CI = 11.4% to 21.5%) in May 2023. It rose more modestly among older age groups, from 0.5% (95% CI = 0.3% to 0.8%) to 7.2% (95% CI = 5.4% to 9.7%) among 25-44-year-olds, and from 0.1% (95% CI = 0.001% to 0.2%) to 2.1% (95% CI = 1.3% to 3.4%) among those aged 45 or older. Similar trends were found when examining the prevalence of daily disposable vaping (Fig. 1B). Prevalence estimates across all adults and stratified by social grade are shown in Supplementary Figure S1.

**Fig. 2:**
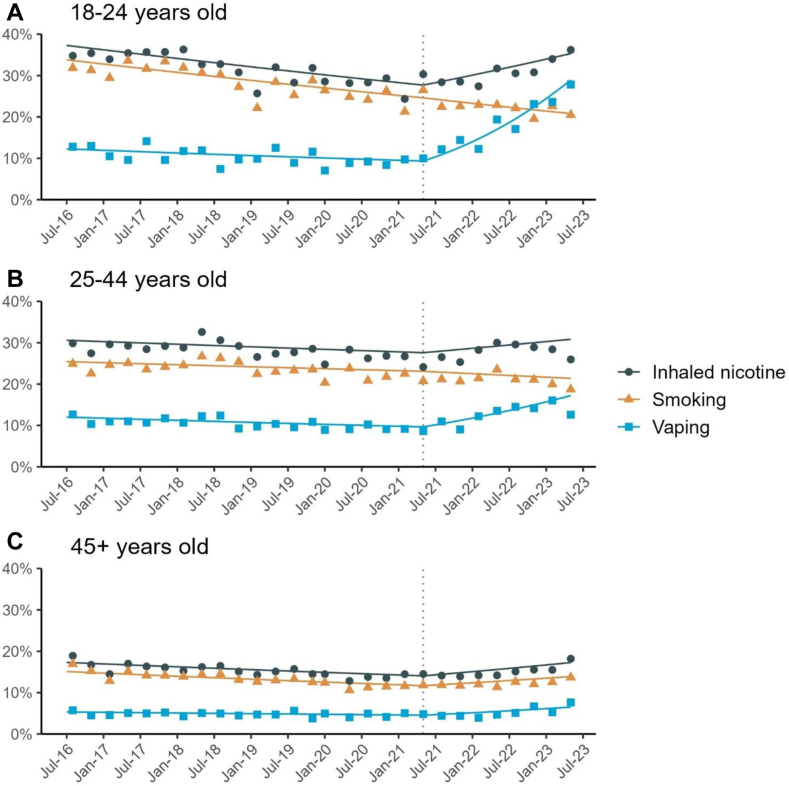
**Prevalence of current inhaled nicotine use, smoking and vaping pre and post the rapid growth of disposable vaping in England by age, July 2016 to May 2023.** Points represent weighted (unmodelled) quarterly prevalence estimates. Lines represent weighted point estimates from a set of logistic regressions, stratified by age and product, with pre- and post-disposables trends modelled linearly. A 0/1 indicator variable was also added as a covariate to account for effects of COVID-19 including the change in modality from face-to-face interviews before to telephone after March 2020, and pre-pandemic lines and points are adjusted to account for this change. The vertical dotted line shows the assumed interruption separating pre- and post-disposable periods.

Results from interrupted time-series models are shown in Table 2, with trends shown visually in Fig. 2. These results show that, in the pre-disposables period, the prevalences of vaping, smoking and inhaled nicotine use were stable or trending downwards over time across all age groups. For vaping, these trends reversed post-disposables; vaping prevalence increased across all age groups between May 2021 and May 2023, with the most rapid increases in the youngest age group. The odds that an 18-24-year-old reported current vaping increased by 99% per year (OR = 1.99; 95% CI = 1.71 to 2.31) across this period, compared with 39% (OR = 1.39; 95% CI = 1.26 to 1.52) in 25 to 44-year-olds and 23% (OR = 1.23; 95% CI = 1.12 to 1.35) in those aged 45 or older. The odds of current inhaled nicotine use (smoking and/or vaping) also increased by an estimated 18% per year (OR = 1.18; 95% CI = 1.05 to 1.33) in 18-year-olds and 15% in those aged 45 or older (OR = 1.15; 95% CI = 1.09 to 1.22). Odds of smoking continued to decline in the younger age groups post-disposables (18-24-year-olds: OR = 0.88, 95% CI = 0.77 to 1.00; 25-44-year-olds: OR = 0.93, 95% CI = 0.86 to 1.00), but increased by an estimated 12% per year in those aged 45 or older (OR = 1.12, 95% CI = 1.05 to 1.20). This increase in smoking occurred even when categorizing people aged 45 or older into more granular groups (Supplementary Table S1). Relative increases in vaping were even greater among those who had never regularly smoked cigarettes (e.g., 18 to 24-year-old never smokers: OR = 2.50, 95% CI = 1.82 to 3.43; Table 3).

**Table 2:** Interrupted time series results showing yearly trends in inhaled nicotine use, smoking and vaping prevalence pre and post the rapid growth of disposable vaping in England.

	Pre-trend^a^		Post-trend^a^		Change in trend	
	OR	95% CI	OR	95% CI	OR	95% CI
**Current inhaled nicotine use (smoking and/or vaping)**						
18 to 24 years	0.91	0.86 to 0.97	1.18	1.05 to 1.33	1.29	1.13 to 1.49
25 to 44 years	0.97	0.93 to 1.01	1.06	0.99 to 1.14	1.10	1.01 to 1.19
≥45 years	0.94	0.91 to 0.98	1.15	1.09 to 1.22	1.22	1.14 to 1.31
Overall	0.95	0.93 to 0.98	1.11	1.06 to 1.16	1.17	1.11 to 1.23
**Current smoking**						
18 to 24 years	0.91	0.85 to 0.97	0.88	0.77 to 1.00	0.97	0.83 to 1.12
25 to 44 years	0.98	0.94 to 1.02	0.93	0.86 to 1.00	0.95	0.87 to 1.05
≥45 years	0.93	0.90 to 0.97	1.12	1.05 to 1.20	1.20	1.11 to 1.30
Overall	0.95	0.93 to 0.97	0.99	0.94 to 1.04	1.04	0.99 to 1.10
**Current vaping**						
18 to 24 years	0.94	0.84 to 1.05	1.99	1.71 to 2.31	2.11	1.72 to 2.59
25 to 44 years	0.95	0.89 to 1.02	1.39	1.26 to 1.52	1.46	1.29 to 1.65
≥45 years	0.97	0.91 to 1.02	1.23	1.12 to 1.35	1.27	1.13 to 1.43
Overall	0.96	0.92 to 1.00	1.44	1.36 to 1.54	1.51	1.39 to 1.63

OR, Odds ratio; CI, Confidence interval.

a Pre-trend shows the yearly relative change in odds of current inhaled nicotine use, smoking, or vaping from July 2016 to May 2021. Post-trend shows the yearly change from May 2021 to May 2023. Results come from weighted logistic regression with gender, social grade, and alcohol consumption included as covariates. A 0/1 indicator variable was also added as a covariate to account for effects of COVID-19 including the change in modality from face-to-face interviews before to telephone after March 2020.

**Table 3:** Interrupted time series results showing yearly trends in vaping prevalence among never smokers pre and post the rapid growth of disposable vaping.

	Pre-trend^a^		Post-trend^a^		Change in trend	
	OR	95% CI	OR	95% CI	OR	95% CI
**Current vaping among never smokers**						
18 to 24 years	1.21	0.87 to 1.70	2.50	1.82 to 3.43	2.06	1.23 to 3.46
25 to 44 years	0.91	0.73 to 1.14	2.01	1.48 to 2.72	2.20	1.47 to 3.29
≥45 years	1.05	0.86 to 1.29	1.48	1.00 to 2.18	1.40	0.88 to 2.23
Overall	0.96	0.92 to 1.00	1.43	1.35 to 1.53	1.50	1.38 to 1.62
**Daily vaping among never smokers**						
18 to 24 years	1.21	0.79 to 1.84	2.25	1.32 to 3.85	1.86	0.87 to 3.96
25 to 44 years	0.94	0.73 to 1.22	1.96	1.33 to 2.88	2.07	1.26 to 3.39
≥45 years	1.16	0.92 to 1.45	0.87	0.34 to 2.20	0.75	0.28 to 1.99
Overall	1.05	0.89 to 1.24	1.99	1.48 to 2.67	1.89	1.32 to 2.71

OR, Odds ratio; CI, Confidence interval.

a Pre-trend shows the yearly relative change in odds of current inhaled nicotine use, smoking, or vaping from July 2016 to May 2021. Post-trend shows the yearly change from May 2021 to May 2023. Results come from weighted logistic regression with gender, social grade, and alcohol consumption included as covariates. A 0/1 indicator variable was also added as a covariate to account for effects of COVID-19 including the change in modality from face-to-face interviews before to telephone after March 2020.

A similar pattern of results was found when restricting the outcomes to daily use (Supplementary Table S2) and when shifting the interruption time-point later by one or two months (Supplementary Table S3). The prevalence of non-disposable vaping also increased among 18 to 24-year-olds since disposables started becoming popular (OR = 1.25, 95% CI = 1.05 to 1.50), but stayed relatively stable among those from older age groups (Supplementary Table S4). For all above analyses, comparable post-disposables trends were found when including data from across Great Britain (including Scotland and Wales) rather than England alone (https://osf.io/z3hgc/).

Results were also similar when we examined changes in outcomes associated with the prevalence of disposable vaping modelled as a continuous variable rather than an abrupt interruption. In such models, we found that for each 1 percentage-point increase in disposable vaping prevalence, current vaping prevalence (in the subsequent month) rose by an estimated 1.07 percentage points (95% CI = 0.96 to 1.19) while the prevalence of inhaled nicotine use also rose by 0.59 percentage points (95% CI = 0.44 to 0.76). There was no strong positive or negative association between disposable vaping and smoking prevalence (*B* = −0.02, 95% CI = −0.17 to 0.12). Analogous patterns were found when stratifying by age group, with no substantial evidence of autocorrelation (Supplementary Table S5).

General patterns were similar after stratifying primary segmented regression models by gender (Supplementary Table S6) and social grade (Supplementary Table S7); across all strata, the youngest age group (18-24-year-olds) saw the greatest rises in current vaping and current inhaled nicotine use.

## Discussion

### Summary

Before June 2021, the prevalence of inhaled nicotine use — both smoking and vaping — had been stable or declining across all age groups in England. However, since the rapid rise in popularity of disposable e-cigarettes, there has been an associated increase in the prevalence of vaping across adults of all ages, with most pronounced rises among the youngest adults. An 18 to 24-year-old's odds of vaping rose by 99% per year since June 2021 (vaping prevalence: 9% in May 2021 to 29% in May 2023), versus 39% in 25 to 44-year-olds (prevalence: 10% to 17%) and 23% in those aged 45 or older (prevalence: 5% to 6%). This increase was not only observed among those who currently or previously smoked; there was an even greater relative rise among people who had never regularly smoked (including a 148% per year increase in odds of vaping among 18 to 24-year-olds who had never regularly smoked). Despite a tripling in vaping among young adults, smoking continued to decline at the same rate pre- and post-disposables (whereas smoking prevalence may have started to increase in those over 45 years-old). As a result of these changes in vaping and smoking, downward trends in inhaled nicotine use have reversed, and nicotine use now appears to be rising among young adults (18% increase in odds of inhaled nicotine use per year among 18 to 24-year-olds, with prevalence rising from 28% in May 2021 to 35% in May 2023). Nonetheless, when comparing across ages, declines in smoking were most pronounced in age groups (18 to 24-year-olds) with the largest increases in vaping.

### Interpretation

Our results broadly align with what was observed among youth and young adults in the US following the rapid rise in vaping (driven by JUUL) from 2017 to 2019, where there were increases in the proportion of young people using nicotine without a clear *acceleration* in the rate of decline in cigarette smoking.^28^ The interpretation of the continued decline in smoking among young adults (aged 18 to 24 years) in the current study relies on which group one considers to be the appropriate counterfactual; i.e., what would have happened if disposable vapes had not been available. One assumption we could make is that if disposable vapes did not enter the market, smoking prevalence would have continued to fall at the same rate as from 2016 to 2021. If this is the assumed counterfactual scenario, then we see little evidence for an effect, positive or negative, of disposable vapes on young people's smoking. However, another assumption is that there were other events since June 2021 — including potential long-term effects of the COVID-19 pandemic and a cost-of-living crisis — that would have affected the prevalence of smoking among all age groups, even if disposable vapes had not entered the market. Under this counterfactual, to examine the population-level effects of disposable vapes, we must compare the change in trends across age groups with higher or lower adoption of disposable vaping. Doing so we see that in the oldest age group (≥45), among whom disposable vaping prevalence only rose modestly, there was a reversal in downward trends in smoking such that, since June 2021, smoking prevalence has been increasing. Conversely, the youngest age groups (18 to 24, 25 to 44) saw continued declines in smoking. Thus, one may conclude that had disposable vapes not become popular in younger age groups, smoking may have increased. However, this assumes the factors affecting smoking prevalence are similar for both younger and older groups, which may not necessarily be the case.

The rise in inhaled nicotine use among young people since disposable vapes started becoming popular — and the sharp increase in vaping among young never smokers — suggests that these products may be leading those who would otherwise not have used nicotine to take up vaping. One hypothesis is that these products only lead to experimental, non-daily use rather than dependent, daily use. However, our data do not support this: many disposable vapers reported using their product daily, and we saw similar relative increases in the prevalence of daily vaping as in overall vaping.

There was also no evidence that disposable vapes had displaced other types of vapes. In fact, the percentage of 18 to 24-year-olds using non-disposable vapes as their main product *increased* throughout the period where disposable vapes became popular. This could be due to people being initially attracted to vaping through disposables, but later transitioning to rechargeable products.

### Policy implications

Our findings show the introduction of disposable vapes was associated with an increase in overall nicotine use in young adults in the absence of an association with accelerating declines in smoking. Assuming these associations are causal, these findings highlight the need for urgent action targeting the availability and appeal of disposable vapes. At the same time, smoking declines appeared to be most pronounced in age groups with the largest increases in vaping, which suggests the potential for policy trade-offs. Policies should be proportionate and try to balance the risk that excessive restrictions on vaping products may be associated with increases in cigarette smoking (as occurred in US states following vape flavour bans and taxation).^32^^,^^33^

The UK government has announced a plan to ban disposable vapes,^13^^,^^17^ and similar plans have been proposed or implemented in several other countries.^14^^,^^16^ One potential danger with banning disposable vapes is that there are now millions of people using the devices who are likely nicotine dependent, and a complete ban while cigarettes remain legal and widely available may signal to a subset of them that cigarettes are a sensible alternative, which would be deleterious for public health.^15^

The charity Action on Smoking and Health (ASH) has instead proposed that an excise tax (e.g., £5 tax per disposable vape sold), putting vapes behind stop counters, and greater restriction on packaging may help to reduce disposable vape use among young people with lower risk of driving people towards cigarette smoking.^43^, ^44^, ^45^ The latter two policies might also be necessary for non-disposable vapes even if a ban is introduced, given that manufacturers of disposable e-cigarettes that are popular among young people have already started making rechargeable versions of their vapes.

### Strengths and limitations

The study has several strengths. We used data from a large, representative sample of adults in England, which provides generalisable insights into population trends across the country. Moreover, we find similar pattens when treating the introduction of disposable vapes as an abrupt interruption and when examining associations with the prevalence of disposable vaping modelled continuously, showing that results were robust to the specific modelling strategy used.

There were several limitations. Firstly, there were insufficient data from Scotland and Wales — which were only surveyed from October 2020 onwards — to precisely estimate the pre-disposables trend and, thus, the change in trend from pre-to post-disposables across all of Great Britain. Nonetheless, we see similar post-disposables trends in Great Britain as in England (https://osf.io/z3hgc/). Secondly, the survey only included a question about which type of e-cigarette people *mainly* use. The prevalence of disposable e-cigarette vaping would have been greater if we also included individuals who used disposables as a secondary product. Third, the measure of daily nicotine use is not specific to e-cigarettes, but all nicotine replacement products. Thus, it is possible that a subset of the people who were classified as vaping daily were in fact vaping non-daily but using other nicotine products, like patches and gum, every day (although such use is rare). Fourth, the interrupted time series analysis relies on the assumption that, had disposables not entered the market, trends in smoking and vaping would have continued to decline at the same rate (linear on log-odds scale) as they were pre-disposables. Fifth, the way vaping is assessed, as linked to a particular reason among current smokers, may have led to an underestimation of vaping prevalence in this group. However, this underestimation is likely to be small as estimates from the STS generally align with those from other surveys.^46^, ^47^, ^48^ Sixth, we examine population-level associations, which cannot be generalised to individual-level causal effects. Finally, we only examined trends among adults aged 18 or older, not those under the legal age of sale. Nonetheless, data from the ASH Youth Survey show similar trends among those aged 11 to 17; the percentage who currently vaped more than doubled from 2021 to 2023, without accelerated declines in cigarette smoking.^5^

### Conclusion

Prior to June 2021, the UK approach towards e-cigarettes appeared able to encourage adults to switch from smoking to vaping while avoiding excess nicotine use among young people.^49^^,^^50^ However, our data show that since disposables started gaining widespread popularity, the prevalence of vaping has been rapidly increasing among young adults, including among those who have never regularly smoked cigarettes. As a result, downward trends in inhaled nicotine use have reversed in England. Cigarette smoking has increased slightly among older age groups but has continued to decline in 18 to 24-year-olds, the age group with the most pronounced rise in disposable vaping. Urgent action is needed to curb the rise in vaping among people who would otherwise avoid nicotine entirely. This could include an excise tax, packaging restriction, and putting vapes behind shop counters. Plans to ban disposable vapes have also been announced in the UK and elsewhere. However, policies must avoid signalling that cigarette smoking — the most harmful form of nicotine use — is a better alternative.

## Contributors

HTB led the formal analysis. HTB and SJ wrote the original draft of the manuscript. SJ, JB, EB, and LS also contributed to writing the manuscript (reviewing and editing). Authors HTB and JB directly accessed and verified the underlying data.

## Data sharing statement

Data are available upon request, and analysis code is openly available online (https://osf.io/z3hgc/).

## Declaration of interests

HTB and SJ declare no conflicts of interest. JB has received unrestricted research funding to study smoking cessation from manufacturers of smoking cessation medications (Pfizer; Johnson & Johnson). LS has received honoraria for talks, unrestricted research grants and travel expenses to attend meetings and workshops from manufactures of smoking cessation medications (Pfizer; Johnson & Johnson), and has acted as paid reviewer for grant awarding bodies and as a paid consultant for health care companies.
