# Supplementary material for: Trends in vaping and smoking following the rise of disposable e-cigarettes: a repeat cross-sectional study in England between 2016 and 2023
Source: Lancet Reg Health Eur. 2024 May 23;42:100924. doi: 10.1016/j.lanepe.2024.100924 (PMC11281926; doi:10.1016/j.lanepe.2024.100924)
Supplement: Supplementary Figure and Tables [file mmc1.docx]

**Supplementary Table 1: Additional older age categories for interrupted time series results showing yearly trends in inhaled nicotine use, smoking and vaping prevalence pre and post the rapid growth of disposable vaping in England.**

|  | Pre-trend* | |  | Post-trend* | |  | Change in trend | |
| --- | --- | --- | --- | --- | --- | --- | --- | --- |
|  | OR | 95%CI |  | OR | 95%CI |  | OR | 95%CI |
| Current inhaled nicotine use (smoking and/or vaping) | | | | | | |  |  |
| 18-24 years | 0.91 | 0.86-0.97 |  | 1.18 | 1.05-1.33 |  | 1.29 | 1.13-1.49 |
| 25-44 years | 0.97 | 0.93-1.01 |  | 1.06 | 0.99-1.14 |  | 1.10 | 1.01-1.19 |
| 45-54 years | 0.94 | 0.89-0.99 |  | 1.28 | 1.16-1.41 |  | 1.37 | 1.21-1.54 |
| 55-64 years | 0.97 | 0.91-1.03 |  | 1.12 | 1.01-1.25 |  | 1.16 | 1.02-1.32 |
| ≥65 years | 0.97 | 0.91-1.02 |  | 1.06 | 0.95-1.17 |  | 1.09 | 0.96-1.24 |
| Overall | 0.95 | 0.93-0.98 |  | 1.11 | 1.06-1.16 |  | 1.17 | 1.11-1.23 |
| Current smoking |  |  |  |  |  |  |  |  |
| 18-24 years | 0.91 | 0.85-0.97 |  | 0.88 | 0.77-1.00 |  | 0.97 | 0.83-1.12 |
| 25-44 years | 0.98 | 0.94-1.02 |  | 0.93 | 0.86-1.00 |  | 0.95 | 0.87-1.05 |
| 45-54 years | 0.93 | 0.88-0.99 |  | 1.21 | 1.08-1.34 |  | 1.29 | 1.14-1.47 |
| 55-64 years | 0.94 | 0.89-1.00 |  | 1.11 | 0.99-1.25 |  | 1.18 | 1.02-1.36 |
| ≥65 years | 0.95 | 0.90-1.01 |  | 1.05 | 0.94-1.17 |  | 1.10 | 0.96-1.26 |
| Overall | 0.95 | 0.93-0.97 |  | 0.99 | 0.94-1.04 |  | 1.04 | 0.99-1.10 |
| Current vaping |  |  |  |  |  |  |  |  |
| 18-24 years | 0.94 | 0.84-1.05 |  | 1.99 | 1.71-2.31 |  | 2.11 | 1.72-2.59 |
| 25-44 years | 0.95 | 0.89-1.02 |  | 1.39 | 1.26-1.52 |  | 1.46 | 1.29-1.65 |
| 45-54 years | 0.95 | 0.87-1.03 |  | 1.34 | 1.15-1.55 |  | 1.41 | 1.18-1.69 |
| 55-64 years | 1.03 | 0.94-1.14 |  | 1.30 | 1.10-1.54 |  | 1.26 | 1.03-1.55 |
| ≥65 years | 0.97 | 0.86-1.09 |  | 1.02 | 0.83-1.26 |  | 1.06 | 0.82-1.36 |
| Overall | 0.96 | 0.92-1.00 |  | 1.44 | 1.36-1.54 |  | 1.51 | 1.39-1.63 |

OR = Odds ratio. CI = Confidence interval.

*Pre-trend shows the yearly relative change in odds of current inhaled nicotine use, smoking, or vaping from July 2016 to May 2021. Post-trend shows the yearly change from May 2021 to May 2023. Results come from weighted logistic regression with sex, social grade, and alcohol consumption included as covariates. A 0/1 indicator variable was also added as a covariate to account for the change in modality from face-to-face interviews before to telephone after March 2020.

**Supplementary Table 2: Interrupted time series results showing yearly trends in daily inhaled nicotine use, daily cigarette smoking and daily vaping prevalence pre and post the rapid growth of disposable vaping in England.**

|  | Pre-trend* | |  | Post-trend* | |  | Change in trend | |
| --- | --- | --- | --- | --- | --- | --- | --- | --- |
|  | OR | 95%CI |  | OR | 95%CI |  | OR | 95%CI |
| Daily inhaled nicotine use (smoking and/or vaping) | | | | | | |  |  |
| 18-24 years | 0.90 | 0.84-0.97 |  | 1.17 | 0.96-1.42 |  | 1.30 | 1.05-1.60 |
| 25-44 years | 0.97 | 0.93-1.01 |  | 0.97 | 0.86-1.09 |  | 1.00 | 0.88-1.14 |
| ≥45 years | 0.94 | 0.91-0.97 |  | 1.14 | 1.04-1.25 |  | 1.21 | 1.09-1.35 |
| Overall | 0.95 | 0.92-0.97 |  | 1.07 | 0.99-1.14 |  | 1.12 | 1.04-1.21 |
| Daily cigarette smoking |  |  |  |  |  |  |  |  |
| 18-24 years | 0.91 | 0.85-0.97 |  | 0.82 | 0.70-0.97 |  | 0.90 | 0.75-1.09 |
| 25-44 years | 0.97 | 0.93-1.01 |  | 0.93 | 0.85-1.02 |  | 0.96 | 0.86-1.06 |
| ≥45 years | 0.94 | 0.90-0.97 |  | 1.14 | 1.06-1.23 |  | 1.22 | 1.12-1.33 |
| Overall | 0.95 | 0.92-0.97 |  | 1.00 | 0.94-1.05 |  | 1.05 | 0.98-1.12 |
| Daily vaping |  |  |  |  |  |  |  |  |
| 18-24 years | 0.92 | 0.80-1.05 |  | 1.91 | 1.47-2.48 |  | 2.08 | 1.53-2.83 |
| 25-44 years | 0.97 | 0.89-1.05 |  | 1.24 | 1.05-1.46 |  | 1.27 | 1.05-1.55 |
| ≥45 years | 0.96 | 0.90-1.03 |  | 1.25 | 1.05-1.47 |  | 1.30 | 1.07-1.56 |
| Overall | 0.96 | 0.92-1.01 |  | 1.36 | 1.22-1.52 |  | 1.41 | 1.25-1.60 |

OR = Odds ratio. CI = Confidence interval.

*Pre-trend shows the yearly relative change in odds of current daily inhaled nicotine use, cigarette smoking, or vaping from July 2016 to May 2021. Post-trend shows the yearly change from May 2021 to May 2023. Results come from weighted logistic regression with sex, social grade, alcohol consumption, and country included as covariates. A 0/1 indicator variable was also added as a covariate to account for the change in modality from face-to-face interviews before to telephone after March 2020.

**Supplementary Table 3: Sensitivity analysis shifting interruption point forward one or two months. Interrupted time series results showing yearly trends in inhaled nicotine use, smoking and vaping prevalence pre and post the rapid growth of disposable vaping in England.**

|  | Pre-trend* | |  | Post-trend* | |  | Change in trend | |
| --- | --- | --- | --- | --- | --- | --- | --- | --- |
|  | OR | 95%CI |  | OR | 95%CI |  | OR | 95%CI |
| *Interruption in July 2021* | | | | | | |  |  |
| Current inhaled nicotine use (smoking and/or vaping) | | | | | | |  |  |
| 18-24 years | 0.91 | 0.86-0.97 |  | 1.19 | 1.06-1.35 |  | 1.31 | 1.13-1.51 |
| 25-44 years | 0.97 | 0.93-1.01 |  | 1.07 | 0.99-1.15 |  | 1.10 | 1.01-1.20 |
| ≥45 years | 0.94 | 0.91-0.98 |  | 1.16 | 1.09-1.24 |  | 1.23 | 1.14-1.33 |
| Overall | 0.95 | 0.93-0.98 |  | 1.12 | 1.07-1.18 |  | 1.18 | 1.12-1.25 |
| Current smoking |  |  |  |  |  |  |  |  |
| 18-24 years | 0.91 | 0.85-0.97 |  | 0.88 | 0.77-1.00 |  | 0.97 | 0.83-1.13 |
| 25-44 years | 0.97 | 0.94-1.02 |  | 0.92 | 0.85-1.00 |  | 0.95 | 0.86-1.04 |
| ≥45 years | 0.93 | 0.90-0.97 |  | 1.13 | 1.06-1.21 |  | 1.21 | 1.12-1.32 |
| Overall | 0.95 | 0.93-0.97 |  | 0.99 | 0.94-1.04 |  | 1.05 | 0.99-1.11 |
| Current vaping |  |  |  |  |  |  |  |  |
| 18-24 years | 0.95 | 0.85-1.06 |  | 2.02 | 1.73-2.37 |  | 2.13 | 1.72-2.63 |
| 25-44 years | 0.95 | 0.89-1.02 |  | 1.42 | 1.29-1.57 |  | 1.49 | 1.31-1.70 |
| ≥45 years | 0.96 | 0.91-1.02 |  | 1.26 | 1.14-1.39 |  | 1.31 | 1.16-1.48 |
| Overall | 0.96 | 0.92-1.00 |  | 1.47 | 1.38-1.57 |  | 1.53 | 1.41-1.66 |
| *Interruption in August 2021* |  |  |  |  |  |  |  |  |
| Current inhaled nicotine use (smoking and/or vaping) | | | | | | | | |
| 18-24 years | 0.91 | 0.86-0.97 |  | 1.21 | 1.06-1.38 |  | 1.32 | 1.14-1.54 |
| 25-44 years | 0.97 | 0.93-1.01 |  | 1.07 | 0.99-1.16 |  | 1.11 | 1.01-1.21 |
| ≥45 years | 0.94 | 0.91-0.98 |  | 1.18 | 1.10-1.26 |  | 1.25 | 1.15-1.35 |
| Overall | 0.95 | 0.93-0.98 |  | 1.13 | 1.08-1.19 |  | 1.19 | 1.12-1.26 |
| Current smoking | | | | | | | | |
| 18-24 years | 0.91 | 0.85-0.97 |  | 0.88 | 0.76-1.01 |  | 0.97 | 0.82-1.14 |
| 25-44 years | 0.97 | 0.94-1.01 |  | 0.92 | 0.85-1.00 |  | 0.95 | 0.86-1.05 |
| ≥45 years | 0.93 | 0.90-0.97 |  | 1.14 | 1.06-1.23 |  | 1.22 | 1.12-1.33 |
| Overall | 0.95 | 0.93-0.97 |  | 1.00 | 0.94-1.05 |  | 1.05 | 0.99-1.11 |
| Current vaping | | | | | | | | |
| 18-24 years | 0.96 | 0.86-1.07 |  | 2.07 | 1.76-2.45 |  | 2.16 | 1.74-2.69 |
| 25-44 years | 0.96 | 0.90-1.02 |  | 1.44 | 1.30-1.60 |  | 1.51 | 1.32-1.73 |
| ≥45 years | 0.96 | 0.91-1.02 |  | 1.28 | 1.15-1.43 |  | 1.33 | 1.17-1.51 |
| Overall | 0.96 | 0.92-1.00 |  | 1.50 | 1.40-1.60 |  | 1.55 | 1.42-1.69 |

OR = Odds ratio. CI = Confidence interval.

*Pre-trend shows the yearly relative change in odds of current inhaled nicotine use, smoking, or vaping from July 2016 to June/July 2021. Post-trend shows the yearly change from June/July 2021 to May 2023. Results come from weighted logistic regression with sex, social grade, and alcohol consumption included as covariates. A 0/1 indicator variable was also added as a covariate to account for the change in modality from face-to-face interviews before to telephone after March 2020.

**Supplementary Table 4: Interrupted time series results showing yearly trends in non-disposable vaping prevalence pre and post the rapid growth of disposable vaping in England.**

|  | Pre-trend* | |  | Post-trend* | |  | Change in trend | |
| --- | --- | --- | --- | --- | --- | --- | --- | --- |
|  | OR | 95%CI |  | OR | 95%CI |  | OR | 95%CI |
| Current vaping non-disposables |  |  |  |  |  |  |  |  |
| 18-24 years | 0.96 | 0.86-1.07 |  | 1.25 | 1.05-1.50 |  | 1.31 | 1.04-1.64 |
| 25-44 years | 0.94 | 0.88-1.01 |  | 1.06 | 0.96-1.17 |  | 1.13 | 0.99-1.29 |
| ≥45 years | 0.98 | 0.92-1.03 |  | 1.05 | 0.95-1.16 |  | 1.07 | 0.95-1.21 |
| Overall | 0.96 | 0.92-1.00 |  | 1.09 | 1.02-1.17 |  | 1.12 | 1.04-1.24 |

OR = Odds ratio. CI = Confidence interval.

*Pre-trend shows the yearly relative change in odds of current non-disposable vaping from July 2016 to May 2021. Post-trend shows the yearly change from May 2021 to May 2023. Results come from weighted logistic regression with sex, social grade, alcohol consumption, and country included as covariates. A 0/1 indicator variable was also added as a covariate to account for the change in modality from face-to-face interviews before to telephone after March 2020.

**Supplementary Table 5: Linear regression results treating monthly prevalence of disposable e-cigarette use as a continuous variable.**

|  | Regression Coefficient* | |  | Autocorrelation  (Durbin–Watson Test) |
| --- | --- | --- | --- | --- |
|  | Beta* | 95%CI |  | *p-*value |
| Current inhaled nicotine use |  |  |  |  |
| 18-24 years | +0.55% | +0.31% to +0.80% |  | .546 |
| 25-44 years | +0.47% | +0.05% to +0.88% |  | .180 |
| ≥45 years | +1.73% | +0.86% to +2.60% |  | .426 |
| Current smoking |  |  |  |  |
| 18-24 years | -0.03% | -0.28% to +0.21% |  | .448 |
| 25-44 years | -0.11% | -0.47% to +0.24% |  | .510 |
| ≥45 years | +1.34% | +0.52% to +2.17% |  | .210 |
| Current vaping |  |  |  |  |
| 18-24 years | +0.98% | +0.79% to +1.17% |  | .622 |
| 25-44 years | +0.82% | +0.50% to +1.14% |  | .232 |
| ≥45 years | +0.73% | +0.22% to +1.25% |  | .146 |

Linear regression models were all run using aggregate monthly data, with outcomes of current inhaled nicotine use, smoking, and vaping prevalence. Predictors were the prevalence of disposable e-cigarette vaping in the previous month, seasonality (modelled using splines), temporal trend (0 in first month, increasing by one in every subsequent month), and an 0/1 indicator variable to account for the change in modality from face-to-face to telephone interview after March 2020. Models were run separately for each age group.

* Beta represents the percentage point change in outcomes for every one percentage point increase in the prevalence of disposable e-cigarette vaping.

**Supplementary Table 6: Gender-stratified interrupted time series results showing yearly trends in inhaled nicotine use, smoking and vaping prevalence pre and post the rapid growth of disposable vaping in England.**

|  | Pre-trend* | |  | Post-trend* | |  | Change in trend | |
| --- | --- | --- | --- | --- | --- | --- | --- | --- |
|  | OR | 95%CI |  | OR | 95%CI |  | OR | 95%CI |
| *Among Women* | | | | | | |  |  |
| Current inhaled nicotine use (smoking and/or vaping) | | | | | | |  |  |
| 18-24 years | 0.94 | 0.86-1.03 |  | 1.36 | 1.15-1.60 |  | 1.44 | 1.17-1.76 |
| 25-44 years | 0.98 | 0.92-1.03 |  | 1.11 | 1.01-1.23 |  | 1.14 | 1.01-1.29 |
| ≥45 years | 0.95 | 0.90-0.99 |  | 1.16 | 1.07-1.26 |  | 1.23 | 1.11-1.36 |
| Overall | 0.96 | 0.93-0.99 |  | 1.15 | 1.09-1.23 |  | 1.22 | 1.13-1.31 |
| Current smoking |  |  |  |  |  |  |  |  |
| 18-24 years | 0.94 | 0.85-1.03 |  | 0.87 | 0.72-1.05 |  | 0.93 | 0.74-1.16 |
| 25-44 years | 0.98 | 0.92-1.04 |  | 0.93 | 0.84-1.04 |  | 0.95 | 0.84-1.09 |
| ≥45 years | 0.94 | 0.89-0.99 |  | 1.11 | 1.01-1.21 |  | 1.18 | 1.06-1.32 |
| Overall | 0.95 | 0.92-0.99 |  | 1.00 | 0.93-1.06 |  | 1.04 | 0.96-1.13 |
| Current vaping |  |  |  |  |  |  |  |  |
| 18-24 years | 1.00 | 0.82-1.21 |  | 2.30 | 1.85-2.85 |  | 2.30 | 1.69-3.14 |
| 25-44 years | 0.94 | 0.86-1.03 |  | 1.57 | 1.37-1.79 |  | 1.66 | 1.40-1.98 |
| ≥45 years | 0.97 | 0.89-1.05 |  | 1.28 | 1.11-1.46 |  | 1.31 | 1.11-1.56 |
| Overall | 0.97 | 0.91-1.02 |  | 1.57 | 1.44-1.71 |  | 1.62 | 1.45-1.82 |
| *Among Men* |  |  |  |  |  |  |  |  |
| Current inhaled nicotine use (smoking and/or vaping) | | | | | | | | |
| 18-24 years | 0.89 | 0.82-0.97 |  | 1.06 | 0.90-1.25 |  | 1.19 | 0.98-1.45 |
| 25-44 years | 0.97 | 0.92-1.02 |  | 1.03 | 0.93-1.13 |  | 1.06 | 0.94-1.20 |
| ≥45 years | 0.94 | 0.90-0.98 |  | 1.14 | 1.05-1.24 |  | 1.21 | 1.10-1.34 |
| Overall | 0.95 | 0.92-0.98 |  | 1.07 | 1.00-1.13 |  | 1.13 | 1.05-1.22 |
| Current smoking | | | | | | | | |
| 18-24 years | 0.89 | 0.82-0.97 |  | 0.87 | 0.73-1.04 |  | 0.98 | 0.79-1.20 |
| 25-44 years | 0.97 | 0.92-1.03 |  | 0.92 | 0.83-1.03 |  | 0.95 | 0.83-1.08 |
| ≥45 years | 0.93 | 0.88-0.97 |  | 1.13 | 1.03-1.24 |  | 1.22 | 1.10-1.37 |
| Overall | 0.94 | 0.91-0.98 |  | 0.98 | 0.92-1.05 |  | 1.04 | 0.96-1.13 |
| Current vaping | | | | | | | | |
| 18-24 years | 0.91 | 0.80-1.03 |  | 1.77 | 1.43-2.19 |  | 1.94 | 1.48-2.55 |
| 25-44 years | 0.96 | 0.87-1.05 |  | 1.28 | 1.12-1.47 |  | 1.34 | 1.12-1.60 |
| ≥45 years | 0.96 | 0.89-1.03 |  | 1.20 | 1.05-1.38 |  | 1.26 | 1.07-1.49 |
| Overall | 0.95 | 0.90-1.01 |  | 1.34 | 1.23-1.46 |  | 1.41 | 1.26-1.58 |

OR = Odds ratio. CI = Confidence interval.

*Pre-trend shows the yearly relative change in odds of current inhaled nicotine use, smoking, or vaping from July 2016 to June/July 2021. Post-trend shows the yearly change from June/July 2021 to May 2023. Results come from weighted logistic regression with sex, social grade, and alcohol consumption included as covariates. A 0/1 indicator variable was also added as a covariate to account for the change in modality from face-to-face interviews before to telephone after March 2020.

**Supplementary Table 7: Social grade-stratified interrupted time series results showing yearly trends in inhaled nicotine use, smoking and vaping prevalence pre and post the rapid growth of disposable vaping in England.**

|  | Pre-trend* | |  | Post-trend* | |  | Change in trend | |
| --- | --- | --- | --- | --- | --- | --- | --- | --- |
|  | OR | 95%CI |  | OR | 95%CI |  | OR | 95%CI |
| *Advantaged (ABC1)* | | | | | | |  |  |
| Current inhaled nicotine use (smoking and/or vaping) | | | | | | |  |  |
| 18-24 years | 0.93 | 0.86-1.00 |  | 1.17 | 1.03-1.34 |  | 1.26 | 1.08-1.48 |
| 25-44 years | 0.90 | 0.85-0.94 |  | 1.22 | 1.12-1.31 |  | 1.36 | 1.23-1.50 |
| ≥45 years | 0.90 | 0.86-0.94 |  | 1.11 | 1.03-1.19 |  | 1.23 | 1.12-1.35 |
| Overall | 0.90 | 0.87-0.93 |  | 1.14 | 1.09-1.20 |  | 1.29 | 1.21-1.37 |
| Current smoking |  |  |  |  |  |  |  |  |
| 18-24 years | 0.93 | 0.86-1.00 |  | 0.95 | 0.82-1.10 |  | 1.03 | 0.86-1.22 |
| 25-44 years | 0.90 | 0.85-0.95 |  | 1.12 | 1.03-1.23 |  | 1.25 | 1.12-1.40 |
| ≥45 years | 0.89 | 0.84-0.93 |  | 1.10 | 1.01-1.19 |  | 1.24 | 1.12-1.37 |
| Overall | 0.90 | 0.87-0.93 |  | 1.08 | 1.02-1.14 |  | 1.21 | 1.13-1.29 |
| Current vaping |  |  |  |  |  |  |  |  |
| 18-24 years | 0.91 | 0.80-1.03 |  | 1.82 | 1.54-2.14 |  | 2.01 | 1.60-2.52 |
| 25-44 years | 0.90 | 0.83-0.98 |  | 1.44 | 1.29-1.60 |  | 1.59 | 1.38-1.84 |
| ≥45 years | 0.94 | 0.87-1.01 |  | 1.22 | 1.09-1.38 |  | 1.31 | 1.12-1.52 |
| Overall | 0.92 | 0.87-0.97 |  | 1.42 | 1.32-1.53 |  | 1.55 | 1.41-1.71 |
| *Disadvantaged (C2DE)* |  |  |  |  |  |  |  |  |
| Current inhaled nicotine use (smoking and/or vaping) | | | | | | | | |
| 18-24 years | 0.90 | 0.83-0.98 |  | 1.19 | 1.01-1.40 |  | 1.31 | 1.08-1.60 |
| 25-44 years | 1.02 | 0.96-1.08 |  | 0.98 | 0.89-1.09 |  | 0.96 | 0.85-1.09 |
| ≥45 years | 0.97 | 0.93-1.02 |  | 1.18 | 1.08-1.28 |  | 1.21 | 1.10-1.34 |
| Overall | 0.98 | 0.95-1.02 |  | 1.09 | 1.03-1.16 |  | 1.11 | 1.03-1.19 |
| Current smoking | | | | | | | | |
| 18-24 years | 0.90 | 0.82-0.98 |  | 0.85 | 0.71-1.01 |  | 0.94 | 0.77-1.16 |
| 25-44 years | 1.02 | 0.97-1.08 |  | 0.83 | 0.75-0.93 |  | 0.82 | 0.72-0.93 |
| ≥45 years | 0.96 | 0.92-1.01 |  | 1.14 | 1.04-1.24 |  | 1.18 | 1.06-1.31 |
| Overall | 0.98 | 0.95-1.01 |  | 0.95 | 0.89-1.01 |  | 0.97 | 0.89-1.05 |
| Current vaping | | | | | | | | |
| 18-24 years | 0.97 | 0.83-1.13 |  | 2.06 | 1.67-2.55 |  | 2.13 | 1.60-2.84 |
| 25-44 years | 0.99 | 0.89-1.09 |  | 1.38 | 1.20-1.58 |  | 1.40 | 1.16-1.68 |
| ≥45 years | 0.99 | 0.91-1.07 |  | 1.25 | 1.09-1.42 |  | 1.26 | 1.07-1.49 |
| Overall | 0.99 | 0.93-1.05 |  | 1.45 | 1.33-1.59 |  | 1.47 | 1.31-1.65 |

OR = Odds ratio. CI = Confidence interval. ABC1 = main household income earner has a professional, managerial or supervisory occupation. C2DE = main household income earner has a routine or manual occupation, is unemployed, or is a state pensioner.

*Pre-trend shows the yearly relative change in odds of current inhaled nicotine use, smoking, or vaping from July 2016 to June/July 2021. Post-trend shows the yearly change from June/July 2021 to May 2023. Results come from weighted logistic regression with sex, social grade, and alcohol consumption included as covariates. A 0/1 indicator variable was also added as a covariate to account for the change in modality from face-to-face interviews before to telephone after March 2020.


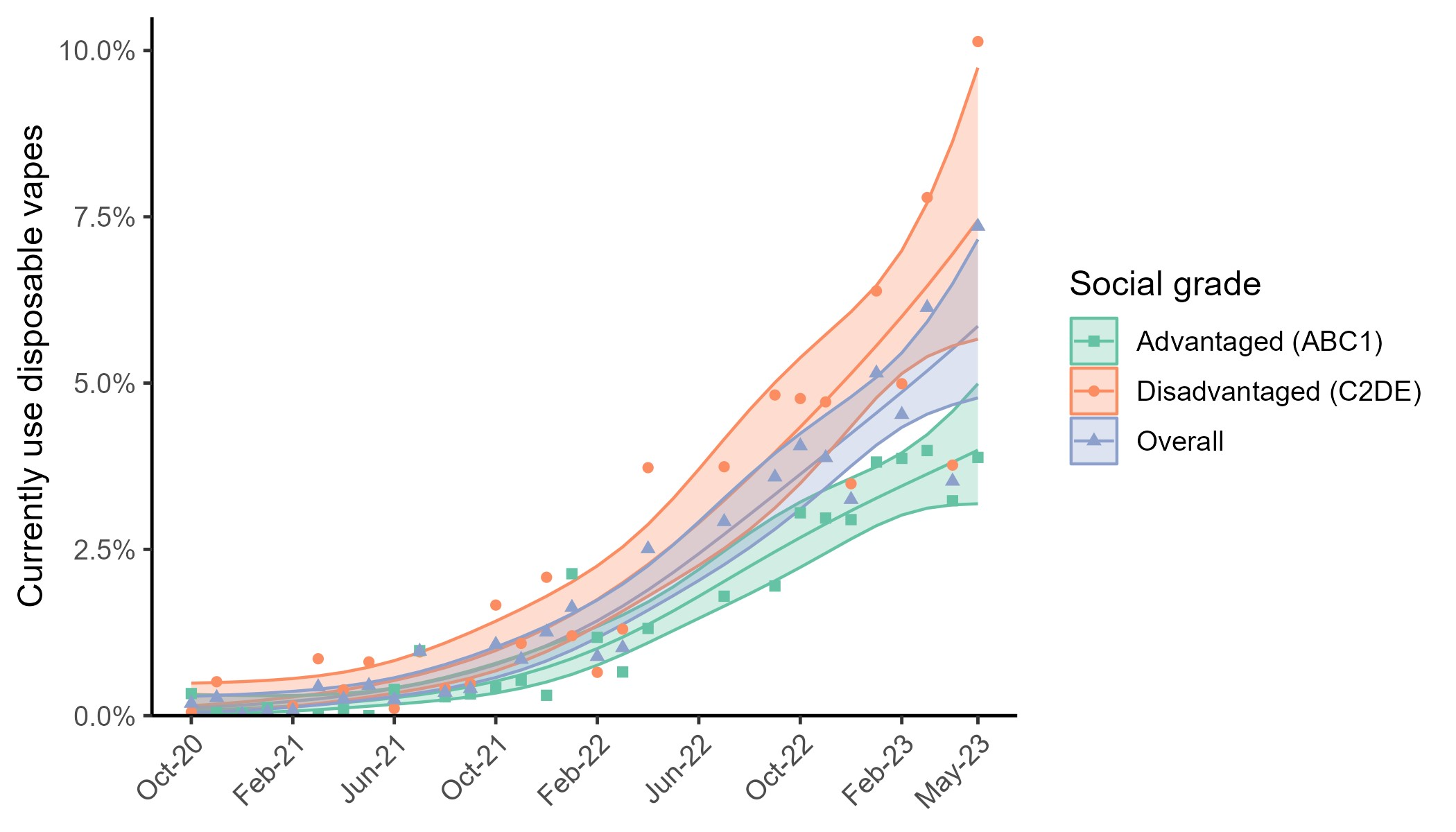
**Supplementary Figure 1: Prevalence of current disposable vaping among adults in Great Britain overall and by occupational social grade, October 2020 to May 2023.** Lines represent weighted point estimates from logistic regression with month modelled non-linearly using restricted cubic splines with four knots, while shaded areas represent 95% confidence intervals.
